# The diagnostic odyssey: insights from parents of children living with an undiagnosed condition

**DOI:** 10.1186/s13023-022-02358-x

**Published:** 2022-06-18

**Authors:** Alicia Bauskis, Cecily Strange, Caron Molster, Colleen Fisher

**Affiliations:** 1 Office of Population Health Genomics, Western Australia Department of Health, 189 Royal Street, East Perth, WA 6004 Australia; 2 School of Population and Global Health, University of Western Australia, 35 Stirling Highway, Crawley, WA 6009 Australia

**Keywords:** Undiagnosed diseases, Rare diseases, Diagnostic odyssey, Diagnosis, Illness experiences, Value of diagnosis, Complex care

## Abstract

**Background:**

People living with rare disease often have protracted journeys towards diagnosis. In the last decade, programs have arisen around the world that are dedicated to ending this ‘diagnostic odyssey’, including the Undiagnosed Diseases Program Western Australia (UDP-WA), which has a focus on finding diagnoses for children and young adults. To explore the lived experience of the diagnostic journey semi-structured interviews were conducted with parents of 11 children at commencement of their involvement in the UDP-WA.

**Results:**

Thematic analysis revealed three main themes that captured parents’ experiences and perspectives. Parents reported (i) the need to respond to significant care needs of their children, which span not only the health system but other systems such as education and disability services. In doing so, parents become the navigator, expert and advocate for their children. Meanwhile, parents are on (ii) the diagnostic odyssey—the rollercoaster of their journey towards diagnosis, which includes various names applied to their child’s condition, and the impact of no diagnosis. Parents described their views on (iii) the value of a diagnosis and the outcomes they expect to be associated with a diagnosis.

**Conclusion:**

Analysis showed an overall significant perceived value of a diagnosis. Our study provides new perspectives on the concept of diagnosis and indicates that parents may benefit from supports for their child’s care needs that are beyond the scope of the UDP-WA.

## Background

Diagnosis is recognised as “central to the practice of medicine” [1 p 9] and has been described as *a starting point, the foundation from which sense-making and experiences are crafted* [2 p 794]. Yet, there are many people living with a disease or condition that does not have a diagnosis, sometimes their entire lifetime. Among these are people living with rare conditions.

Currently there are between 6000 and 8000 different rare diseases globally, with the number of known rare diseases ever growing. While they are individually rare, affecting one in 2000 people or fewer [3] they are collectively quite common. It is conservatively estimated that up to 5.9 per cent of the general population live with a rare disease, equating to an estimated 300 million people worldwide [4]. Rare diseases are often debilitating, life-threatening and tend to affect multiple body systems. Overlaying these physiological features, the person living with a rare disease often faces challenges associated with the direct functional impacts of the condition as well as psychological, financial and a range of other difficulties [5]. They also have to contend with the segmentation of medical specialities, a well-recognised barrier to their multidisciplinary care needs, and encounter challenges accessing supports from other systems, including those which deliver social services [6]. In the Australian context for example, people living with a rare disease often need to access care from across at least two separately funded systems being the medical and disability systems; the latter of which provides care encompassing home care assistance, speech, occupational and physio therapies as well as supports like respite care. Significantly, disease rarity is often associated with diagnostic delay, or no diagnosis at all. Recognition has grown over the last decade of the “diagnostic odyssey”, a term frequently applied in academic literature to describe protracted journeys towards diagnosis for people living with rare diseases [7–10].

A number of survey-based studies have been conducted for both adults and children revealing that the journeys towards diagnosis typically involve multiple interactions with the health system, inconclusive results and misdiagnosis as well as delay in reaching diagnosis [5, 6, 11–13]. For example, Zurynski et al. 2017 [13], showed that 38 per cent of families of children with a rare disease consulted six or more doctors to receive a diagnosis and a survey of adults living with a rare disease revealed that 30 per cent waited more than five years for diagnosis and half had received an incorrect diagnosis [5]. Further, late and misdiagnosis of rare diseases have recognised associations with medical, physical and mental health burdens for the patient, and financial and emotional burden for the patient’s family [10].

Before the term diagnostic odyssey took hold in academic literature the experience of individuals with an unresolved diagnostic status had been studied. Nettleton, 2006 [14] explored the experience of people with ‘medically unexplained symptoms’ (MUS), and found that living with uncertainty, and dealing with a lack of legitimacy and resisting psychological explanations for their condition were significant challenges [14].

Despite studies exploring MUS and the growing body of literature making reference to a diagnostic odyssey for people living with rare diseases, there is a lack of literature that captures the detail of the lived experience of these journeys from the patient and carer perspective. A number of qualitative studies have addressed this in part through a focus on the outcomes of diagnostic (often genetic) investigations for rare diseases in both adults [15] and children [9, 16]. However, until recently, there has been limited literature addressing the experiences of parents of children with undiagnosed conditions, especially with respect to their diagnostic odyssey journey before the outcome of an investigative process is received, and their orientation towards this outcome.

In 2017, Spillmann et al. began to address this significant gap with an article that explored one-page written accounts from parents of children, as well as adults with their own undiagnosed condition, who were applying for the United States of America (USA) Undiagnosed Diseases Program [17]. Spillmann et al. [17], applied Frank’s [18] three illness narrative types of ‘restitution’, ‘chaos’ and ‘quest’ to analyse the accounts. However, as Frank had used these narrative types to explore the narratives of diagnosed adults, they were adapted to capture parental experience of having a child with an undiagnosed condition. In doing so, Spillmann et al. [17] found that all the parents and probands (adults with the condition) described chaos narratives, explaining the emotional challenges and frustrations of continually looking for a diagnosis, with parents particularly concerned with the uncertainty of their child’s future. The authors reported that some of the parents described a restitution narrative of “acceptance of a new normal” [17 p 9] and that several parents provided quest narratives including a focus on the “positive attributes of the child” [17 p 7] and “new parenting strengths and advocacy” [17 p 9]. Spillmann et al. [17] concluded that restitution and quest narratives moderated the chaos parents experienced and found the illness narratives varied between parents and probands.

In addition to the lived experiences surrounding diagnosis, diagnosis itself has been the subject of considerable sociological investigation [19]. Blaxter [1] proposed the domains ‘diagnosis as category’ and ‘diagnosis as process’ with the former in essence being the label or named applied to a disease and the latter being the “negotiation, multiple investigations and trial and error” [2 p 796] undertaken to arrive at the name or label. Blaxter’s domains were expanded by Jutel and Nettleton [2] to include ‘diagnosis as consequence’—the acknowledgement that diagnosis has consequences to those for whom it applies. The three domains of diagnosis as category, process and consequence may be experienced differently by those without diagnostic certainty, warranting further investigation in the context of rare and undiagnosed diseases.

This present study, which reports on interviews conducted with parents of children in a program aimed at finding diagnoses (the Undiagnosed Diseases Program Western Australia, described below), adds significant depth to our understandings of the experiences of parents of children with suspected rare diseases but who remain undiagnosed. Further, the concept of diagnosis is explored in relation to their accounts, helping illuminate elements of these journeys and in turn, providing some new insights into diagnosis.

### The undiagnosed diseases program WA

A significant reason for diagnostic delay is the lack of familiarity among health professionals with the many thousands of rare diseases, an issue compounded by the tendency for rare diseases to display heterogeneity in symptoms, where two individuals with the one rare disease can present very differently. Additionally, assembling the multi-disciplinary teams that are often required to help unlock the cause of multi-system disorders is challenging in fragmented health systems that have silos of medical specialties [20]. All the while, the development and utility of genomics knowledge and technologies increases as the cost of integrating genomics into healthcare systems decreases, a phenomenon that may be accelerated with the substantial investment in genomic technologies during the COVID-19 pandemic. With an estimated 80 per cent of rare diseases being genetic; definitive molecular diagnoses are emerging as potential new answers for those who previously went without a diagnosis [21].

In Western Australia (WA), to help overcome the protracted diagnostic journey and make the most of advances in genomic technologies, a program was set up to provide diagnostic assistance to those with the highest need; children with long standing complex health conditions that, despite extensive efforts by families and physicians, have eluded diagnosis. Based on the USA program of the same name [22], the Undiagnosed Diseases Program WA (UDP-WA) was established in 2016 as a clinical program in the WA public health system with a small amount of seed funding from the WA Department of Health and a reliance on the donated time of a wide range of clinicians. Meanwhile the core staff of the UDP-WA have been enabled through significant philanthropic funding. The WA public health system is exploring ways to make the program sustainable within the system’s funding mechanisms. At present the UDP-WA is currently one of a number of such programs internationally [23, 24]. The UDP-WA accepts patients aged between six months and 16 years, with a paediatric cohort considered as an appropriate starting range due to limited capacity with the possibility to consider expansion later. Eligibility criteria for the program include those who are: (i) generally at least 6 months old; (ii) have chronic, complex and typically multisystem diseases; (iii) are well known to the public health system; (iv) have typically had multiple specialist assessments and hospital admissions; and (v) have clinical factors supporting the possibility of obtaining a diagnosis with current approaches, yet remain undiagnosed [25]

In their 2017 paper, Baynam et al. [25] outlined the seven stages of the UDP-WA program. Critical among these stages is the convening of the UDP-WA multidisciplinary expert panel. This group follows standard operating procedures that includes brainstorming diagnostic approaches (asking questions such as: should we run this scan again?) and possibilities (making suggestions such as: perhaps it is this condition?) for approximately one patient per month, with genomic investigations frequently undertaken and informed by the multidisciplinary process. With the longer-established UDP in the USA achieving approximately 25 per cent diagnostic rate [26], the limited chance of diagnosis is conveyed by the UDP-WA clinical team to families as they enter the program, to help moderate their expectations on the likelihood of diagnosis. At the conclusion of the program (stage 7), all families receive a report, irrespective of whether a diagnosis has been found [25]. The report outlines important new phenotypic findings and test results and suggestions for further assessments and changes in care and can include facilitating connections with relevant support groups or patient communities [25],

## Methods

An evaluation study of the UDP-WA utilising a generic qualitative design [27] aimed at building a greater understanding on families’ journeys as parents/caregivers of undiagnosed children with complex medical conditions, their experience in the program and the impact of diagnosis or any new information that the UDP-WA could provide families. This article reports findings from the baseline parent interview data of the evaluation.

### Participants and recruitment

Parents of children involved in the UDP-WA were invited to be involved in the evaluation study. Invitations were extended by the UDP-WA Coordinator whose role was to liaise with families and coordinate clinical appointments. For those parents agreeing to take part, the Coordinator provided their contact details to the evaluation research team to allow next steps to be arranged. The UDP-WA evaluation team were independent from the UDP-WA care team and participant information shared was restricted to details that enabled the evaluation team to contact families. No other personal information was shared.

Thirteen families were invited to participate with one family declining. In total, thirteen parents (12 families) were involved in the interviews, with one family excluded from the baseline analysis performed for this article as they received a diagnosis before the first interview took place, meaning it did not represent a true baseline. Participants from the remaining 11 families in scope for this article were 11 mothers aged 25 to 50 years and one father (5b) aged 46 years who participated in the same interview as his wife (5a). Despite the interviewer characterising information provided from each individual interviewee (5a and 5b) in this interview as corroborating and supplementing the perspective of the other [28], it may have been that, had individual interviews been undertaken, other information may have been forthcoming reflective of unique individual experiences or perspectives. The children with an undiagnosed condition were aged two to 13 years and their siblings were aged two to 19 years. Eight families had two parents in the primary home. With the exception of one foster mother, all participants were biologically related to their child with an undiagnosed condition. Due to limited patient intake into the UDP-WA, recruitment occurred over an extended period, October 2016 to July 2018.

### Data collection

Data were collected using in-depth, semi-structured interviews. These interviews were conducted with parents at a time and location that suited the family. Two families lived in regional WA and as such, these interviews were conducted by phone. For the nine families based in the Perth metropolitan areas, three interviews took place by phone and the remaining six interviews took place in homes. Interviews were conducted by two of the authors (CS & AB). The interviewers introduced themselves to participants, including their workplace and role, and fostered a rapport and a safe environment for interviewees to discuss their experiences and perspectives. Both interviewers have had several years’ experience working in public health and related community research and CS is a registered nurse. Interview length ranged from 40 min to two hours and 40 min with the majority of interviews being at least one hour in length. The interviewers debriefed following interviews and maintained field notes and an audit trail. All interviews were transcribed verbatim.

### Analysis

A thematic analysis based on the approach proposed by Braun and Clarke [29] was employed with assistance from NVivo11 for data management. Transcripts were read by all authors and the initial thematic analysis involved immersion in the data by authors AB & CS and a generation of initial codes. Noting their different ‘analytical lens’ [27], influenced by a range of factors, significant of which being place of employment, AB & CS discussed their different perspectives and understandings when further revising codes and the development of potential themes and a thematic map [29].

CF and CM undertook a key role in reviewing codes and potential themes. Importantly, their analytical lens [27] was different in terms of their relative ‘distance’ from the data. AB and CS had developed a ‘closeness to data’ [30] through not only hearing interviews first-hand but also through engaging in pre and post interview discussions with participants, which on occasion could lead to spending over three hours in a participant’s home. As such, certain knowledge and assumptions were held by AB and CS, that CM and CF drew out and challenged through both desktop review of data and in–person discussion. These review processes helped the team arrive at final themes and sub-themes, as well as the supporting quotes from participants.

An additional approach taken to understanding and presenting data was that a meaning-centred or interpretive approach to exploring the subjective lived experience of parents with children in the UDP-WA, particularly in terms of their feelings, experiences and perceptions of diagnosis (or lack thereof). In doing so, metaphors parents used to describe their experiences have been incorporated into our results.

## Results

**Fig. 1:**
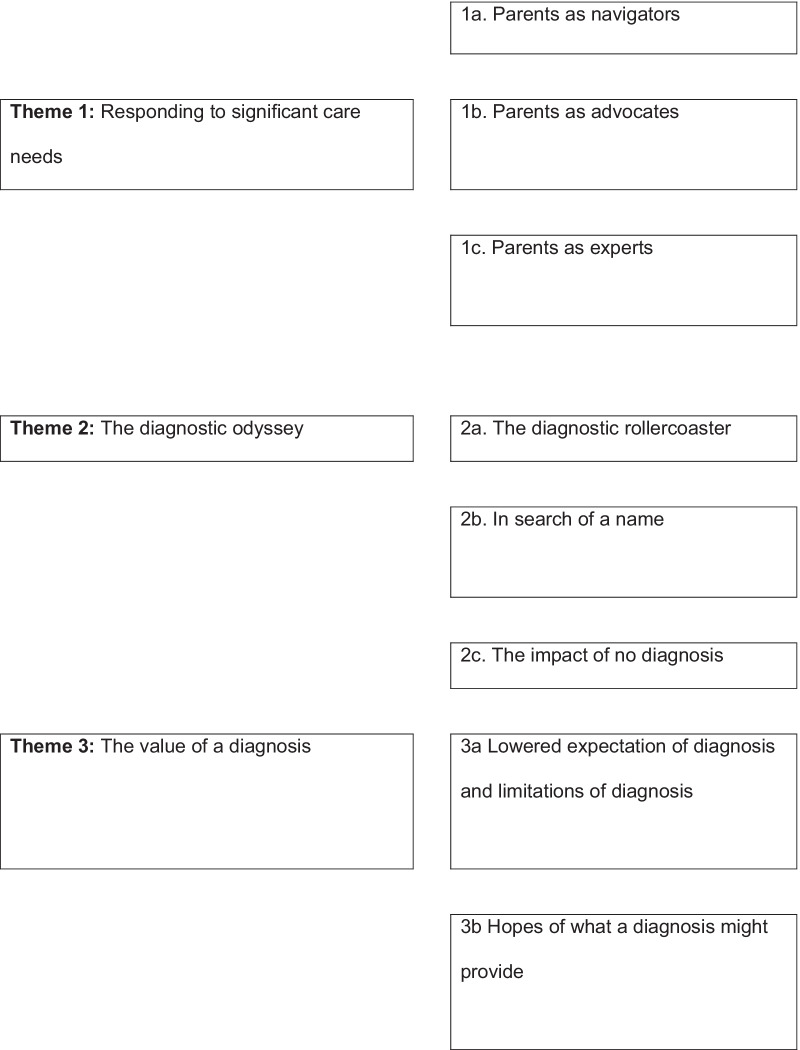
Thematic Map: Experiences of parents with a child who has an undiagnosed condition at the point of commencement with the UDP-WA

Three main themes captured parents’ experiences and perspectives. Parents reported (i) the need to respond to significant care needs of their children, which span not only the health system but other systems such as education and disability services.^1^ In doing so, parents become the navigator, expert and advocate for their children. Meanwhile, parents are on (ii) the diagnostic odyssey—the rollercoaster of their journey towards diagnosis which includes various names applied to their child’s condition, and the impact of no diagnosis. Parents described their views on (iii) the value of a diagnosis and the outcomes they expect to be associated with a diagnosis. Themes and sub-themes are displayed in Fig. 1 below.

### Theme 1: responding to significant care needs

Parents shared their experiences of looking after a child not only without a diagnosis for their condition but often with significant care requirements. On the medical front, many children had multiple symptoms, and many had comorbidities and health conditions requiring frequent attendance at hospital clinics. Several had spent significant periods admitted to hospital. All children saw multiple specialists to help manage their health care with one parent reporting that their child saw 17 different medical specialists in the last year alone.

The range of disability for children, as described by their parents, extended from requiring some support in the mainstream school system to intensive specialist support in an additional needs school. Nearly all parents reported that their child had experienced developmental delays in areas such as speech, feeding and toileting compared to their age equivalent counterparts, with a number requiring intensive supports. These delays varied from being minor such as *maybe a month behind (P2)* through to significant with one parent describing the receipt of *a school psychology report that says something to the effect of the child’s physical and intellectual disability appears to be so profound, it’s difficult to complete this* assessment *(P5a).*

Several parents had experienced the near-loss of their child and for some, concern about their child’s mortality was expressed with one parent describing time with their child as *borrowed* (P11) and another talking about the need to *mak[e] the most of our time as a family together (P7).*

For many parents, concern was first raised about the health of their child before birth or in the first year of life, and they had been navigating the health care system and other support systems thereafter. To meet the care needs of their children, parents described constantly coordinating and managing medical, other support appointments and connections across numerous services. In addition to getting appropriate medical care, children required support with finding a range of services delivered in the community setting such as education and a range of disability services which provided occupational, physio and speech therapy and support, such as respite care.

Several parents spoke about the “system” separation between medical management and disability management when the two are closely connected and would benefit from being managed together rather than in silos.

*Young children, their disability and their medical complexity kind of rest hand in hand… and the system spends its life trying to separate those things…so the systems then need to change to support that better. (P5a)*

Parents’ description of navigating systems invariably included anecdotes of being an advocate for their child. One parent described her role as a *secretary*, as distinct from a caregiver, in navigating both health and disability services and advocating for their child by following up overlooked appointments and trying to secure care support items.

*The care was one thing… but it’s… following up medical professions, because—I haven’t had an appointment for over a year. So, now I have to keep a diary to make sure that he’s getting all his appointments. This morning I’ve been on the phone to the disability services, because they're saying they won’t give us another sleeping system…. And I’ve just been on the phone talking to them to find out why. So, (child) needs his own secretary, as well as a mum. (P6)*

Most parents had to advocate to get their child support within mainstream schooling or access to schools for children with additional needs. Some parents reported having very supportive schools, while others needed to advocate along with the school to secure assistance for their child.

*But in the start, there was a real, for me, a bit of burnout because I was going down the school and I was rattling cages trying to get help and support because he clearly needed it… but no one would deem him worthy of any assistance. I’ve hit my head against brick walls everywhere and got absolutely nowhere. (P12)*

Parents described how they needed to be assertive or proactive in getting the best possible care for their child across all settings. This included developing greater confidence in their ability to understand their child and seek other expert medical opinions if need be.

*I’ve been in the system for a long time. I’ve learned that if I wanted another opinion… I go get it because I’m not happy just taking what’s being told to me… also from the very beginning, if I have an instinct about something, I try and stick to that… I’ve learnt from my mistakes. (P4)*

In addition to navigating and advocating within health and across different systems, parents become experts in their child’s condition and management. Many described needing to convey complex information about their child’s medical condition to clinicians. For one parent this involved filming their child’s seizures never-before seen by their medical team.

For some parents their roles as navigator, advocate and expert extended into proposing investigations or diagnostic possibilities for their child with one parent describing the *self-diagnosis* of an aspect of their child’s condition:

*I found like dyspraxia and things like that and we went into the paediatrician and said, ‘Well, this describes him. Why isn’t it this?’ and then they went, ‘Oh yeah, he does.’ So, you find you’ve got to do a lot on your own self-diagnosis to get anywhere. (P12)*

Another parent, who had undertaken a lot of their own research, explained:

I went to our doctor and said ‘Can we please be referred to genetics? I found some research and I think that there is permutation on this protein.’ (P11)

Despite, or perhaps because of these efforts, most parents spoke of discovering new perspectives on life and experiencing personal growth. Parents talked about sibling maturity and the strength of family members.

We’ve grown compared to what we used to be, like, you know, going through what we did and I think it just makes us bigger people, stronger people. (P2)

Several parents were involved in support groups associated with a feature of their child’s condition such as epilepsy. However, many support organisations are condition-specific and often parents of undiagnosed children are unable to connect to these groups. Belonging to a support group strengthened personal agency and afforded parents a sense of solidarity as well as opportunities to further develop their expertise on their child’s condition and the systems they needed to navigate to meet their child’s needs.

*We (in the support organisation) were all on the same page… you’re already reeling from the fact that your child is no longer normal anymore, and, ‘Okay, so what do we do?’ and nobody can tell you what you got to do. … you’re just surrounded in unknown and when you get to a place like… the support group, everybody doesn’t know everything there, you know. So if you find out one snippet of information, you share it with the group. (P5b)*

### Theme 2: the diagnostic odyssey

The parents involved in this study had children with suspected rare diseases, but they had as yet not received a diagnosis at the time of the interview. As such, they did not have a ‘name’ that they could apply to the full suite of their child’s symptoms. For many parents, concerns about their child’s health were raised during pregnancy, at birth or during the first year of infancy, with several undiagnosed children now in early adolescence. The diagnostic journey commonly involved a *rollercoaster* of concerning and reassuring indicators, including receiving incorrect diagnoses or being informed of potential diagnoses of severe conditions that were later negated. Emotional states experienced by parents during this period included *shock*, *stress*, *worry*, and feeling *overwhelmed.* The emotional rollercoaster experienced with a potentially severe diagnosis is illustrated here:

*So, when I researched into that (potential diagnosis), I got a massive shock, because obviously, this is a severe intellectual disability, where we knew something was wrong, but not to that extent. (later) He’s been tested for all three of them (potential diagnoses) and were negative on all three. So… we kind of went back to the drawing board. (P6)*

Another parent spoke of her experience during pregnancy where she was informed of a diagnosis of a life-limiting condition for which her child would have a life expectancy of several days to under one year, only to be told later that her baby was *fine*.

*They told us that (child) wasn’t gonna survive… that she was (name of chromosomal condition)…where they don’t live longer than a year or they’ll live a couple of days and pass on. And we were thinking, ‘well… would it be okay if she’s not gonna survive or have we come to terms with that?’. And then a week or two weeks later they said ’Oh, no she doesn’t have that’… when they did the amniocentesis… it came back negative. The doctor was like ‘Well, I don’t know what’s wrong with your baby’. One minute they’re saying she’s not gonna live and then she’s (doctor) saying she’s fine… She’s still growing, we just have to wait. (P2)*

Without a diagnosis, signs and symptoms were sometimes elusive and hard for health professionals to pinpoint or to plan management. Children often experienced severe symptoms without warning or explanation contributing to the ‘rollercoaster’ experience, as described here by one parent.

*They’re big seizures and… it was quite unusual, so we were told for a child to present with the type of seizures he was having without some kind of cause, and he was… treated as if he had an infectious disease which they discounted. … he ended up in the ICU and had a variety of drugs pumped into his little body. (later) (P5a)*

Throughout these journeys parents described accounts where the medical professionals were baffled and surprised by children’s presentations yet ultimately agreed that the children were unwell. However, some parents felt that their account of symptoms was not always believed by the medical profession unless it could be witnessed by them first-hand.

*They [medical professionals] captured a clinical seizure on EEG in video and she (the doctor) actually raced down that day and said, ’It’s exactly as you have been describing.’ I said, ’Well, I don’t make it up…’. (P5a)*

While not in possession of an overarching diagnosis, most undiagnosed children had received one or more diagnoses for certain aspects of the condition. These diagnoses, many of which are often considered a complete diagnosis for other individuals, include epilepsy, vasculitis, and autism. They also include generic diagnoses such as intellectual disability, which does not point to a particular condition and includes diagnoses that are both generic and time-limited such as global development delay which is recognised until age six years (in Australia).

*Until the age of six, we’ve been known as global delay. And now he’s turned seven, it’s been classed as intellectual with no name because—yeah, nothing’s been confirmed.” (P6).*

These “labels” can provide a practical function for parents, supporting their communication with service providers and in some cases enabling access to specific supports. *We have got some diagnoses. So, we get support through those. (P11)* With some diagnoses acknowledged as being provided purely to access support:

*When he was at [education support school] he got given the diagnosis of an intellectual disability. Now… he’s only got that label (diagnosis) to keep his spot there. (P9)*

The outcome of their protracted, winding diagnostic pathways that have yet to result in a conclusive, overarching diagnosis, is that parents are still searching for a *name* that they can apply to the full suite of their child’s symptoms, a *label* that means they can *tick a box* to say what their child’s condition was. For several parents this was *“the hardest thing—not having a label to put on it” (P10)* and ultimately, not having a name/diagnosis had a cascade of consequences for parents and their children.

Parents talked of the difficulties they had interacting with the health system when their child did not have a diagnosis. A key issue of concern was the need to repeat their child’s symptoms all the time.

*So, when you’ve got a kid with a laundry list a mile long of issues and no name for it, no diagnosis, you can’t just go in and say, ’My son has this’. You’ve got to list the 50 things that are different about him every time with a different doctor and start again and you never get anywhere. So while the doctors might say this, just give it a name. It does make a difference to be able to just go to school or whatever and say, ’He has this’, and that’s something they can look at and that’s something I can look up. So that was the most challenging thing. (P12)*

Parents described concerns about treatments and healthcare management approaches that had unknown effectiveness. Healthcare teams undertook procedures with no guarantee of usefulness as illustrated here by one parent who described treatments for her child as *grasping at straws* as *they (medical team) have no idea*.

*Because with (child)… it was just trial and error with treatments to try and keep him alive because things that they were expecting to work didn’t work and actually went quite often the opposite way. (P11)*

*Nobody seemed to be able to say to us, ‘This is what we’re gonna do and this is what is going to work.’ Like when he had his first surgery… they said it went great… and then they said ‘Well…we don’t know whether his [body organ] is just gonna shut down at one point and he’ll pass away.’ Because they have no idea. So… that’s been the hardest thing… I kind of felt that we were fumbling along just hoping that something would work without anyone being able to say ‘In this condition, if you do this, this is what will happen.’ (P11)*

With no diagnosis or clear clinical pathway parents felt there was *a black hole (they) fell into very early as there was no box to tick. (P5a)* The health care system was responding to changes but was not able to predict or plan for future unknown needs. Several parents described how new and unexpected health issues continued to present.

*Our whole roller coaster from when she was discharged after the diaphragmatic hernia repair has been these shocks…When she was discharged… I was told that she was fixed. So, from then on, it was just these really stressful situations, where she'd start doing something that's completely out of the blue…Why is she shaking? Why is she turning blue?…I’d take her to hospital, ‘So, she's not tolerating her food’… ‘Give her a nasogastric top-up… go home’. Then I'd be giving her the nasogastric top-up, but she's bringing that all up… Back to hospital… And then, I was coming home with a child that I knew… there’s something bigger. There's so many things wrong with (child) and I still don’t know why. (P7)*

Without a diagnosis, many parents felt they did not know what the future will hold for their child and this was concerning to them with one parent describing that *I just want to know what it is because I wanna know what the future holds. (P11).*

*We…never will know if (child) will ever catch up to the same level of his peers. He’s at age three level now. Will he ever catch up? It’s just not knowing what to expect for him when he’s older. I guess with some syndromes, you’ve got an idea. We—that’s the hardest thing—is not having a label to put to it and just not knowing what the future holds for him—I mean… he’s a happy bright little boy and very sociable… but who—intellectually, we don’t know where it’s gonna head... And that’s the thing that’s eating us up, really. We just don’t know where life is gonna—you want the best for your children. (P10)*

As mentioned in Theme 1, some parents belonged to a support group for one of the health conditions their child had, for example, epilepsy. However, for other parents, without any diagnoses for their child it was difficult to connect with others to share experiences and find support.

*No support network in a sense. I can’t find another family with a child like (him) that I can connect with and have that connection… ‘My child has this, and I can’t connect with you because my child doesn’t have that.’ He’s got A, B, C, D, E… so I can’t connect with anyone. I’m on support pages but I haven’t come across anyone that has any similarity to (child) that I can connect with. (P9)*

### Theme 3: the value of a diagnosis

To help moderate expectations, UDP-WA personnel prepare parents of children going through the program by explaining that the chance of finding a diagnosis is about one in four. This may have contributed to the fact that most parents showed a degree of restraint when talking about diagnosis and their child. Additionally, years of unsuccessful investigations had lowered the expectations of some for finding a diagnosis, as described here.

*I don’t mean this the way it sounds, but I don’t have big expectations because it’s been this long and they still don’t really know what’s going on with him. (P10)*

Most children in this study were living with very complex medical conditions and some parents candidly shared that a diagnosis would not alter their child’s health situation. As described by one parent*, it’s not gonna fix (child*) (P10). Another described that a diagnosis would unlikely result in any health improvements, or change the life trajectory of their child, nor that of their own:

*We’re really realistic. It doesn’t matter—and the answer… it won’t make any difference to our journey or (child’s) life because it's unlikely to change. (P5a)*

Generally, parents had established careful boundaries around their expectations of the likelihood of a diagnosis and what it may deliver, and did not express apprehension about receiving a diagnosis. In fact, all parents unequivocally felt that there were real benefits in their child being diagnosed. Generally, parents felt that obtaining a diagnosis or new information could provide answers or *context* to help explain and understand their child and *what they are dealing with*. This would lead to greater certainty about the future and enable the development of plans or health care pathways to follow, although a diagnosis was not expected to change the daily health needs and care of their child. As proposed by one parent, the knowledge that diagnosis brings is *power*:

*Knowledge is power, like I said before. And for me, tell me what I'm dealing with, because it is what it is. It's not gonna change anything, it's not gonna make her any sicker, it's just gonna tell me what I can expect down the track. And every doctor that I see, when I talk about prognosis, or even when some new thing comes up… Every single time I see a doctor, ’What was the overarching condition? What are we dealing with?’. They don’t know, so there’s no information that I can give them to help them put… what's happening with her, into a context, either. So, it's not just about me and my little research as a very medically untrained person, it's also about the doctors, and giving them a context. (P7)*

A number of parents saw the possibility that a diagnosis could help predict future health problems of their child, enabling carers to *know what to look out for*:

*And in my mind, one of the other main reasons I want a name is that sometimes what comes with the diagnosis is knowledge that they might be prone to heart problems or something else, just to know what to look out for but it all probably surrounds understanding and getting support for him. (P12)*

Some parents talked about how a diagnosis will help them plan future pregnancies and clarify the risk of reoccurrence for the siblings when they have children. A diagnosis would provide some information or reassurance on the risk, if any, as described here:

*The geneticist said ‘Are you gonna have more children?’ and we’re like ‘No’ and she goes ‘The testing is still relevant because of (siblings)’. She said, ‘When they have children, they may need to know this information,’ and I was like, ‘Oh! I never thought about my children having children.’ (P1)*

Others spoke of reduced stress in their lives that diagnosis might bring. One parent was seeking a diagnosis for this purpose, when previously they would have avoided the opportunity for their child to be diagnosed.

*I almost would’ve said no to genetic testing at the beginning… I didn’t want to know there was something wrong with my child. I just wanted it not to be so… I just lived in denial… eventually she’ll get better and she’ll be fine. Whereas now… I’ve sort of come full circle and it’s like… if I now know what’s going on hopefully that is going to relieve some of the stress. (P1)*

Several parents spoke of the potential benefits of connecting with other families with a child with the same diagnosis. Connecting with families of children with the same diagnosis would help with explaining what is currently unknown about their child, such as some of their features and characteristics.

*We can connect through stories and what we’ve gone through and if they’ve tried something different, and just to have a little bit more explanation like why he was…so short in height because no one has been able to explain that. So… it’s just little things. (P9)*

For other parents, connections with other families with children with the same condition might help to reduce future impact as described here:

*We can’t be the only child in the world that has got all these things with their brains going wrong. (later) but if there’s another child in the world that’s got it… we might be able to get in contact with them and see what they do to try and minimise any impact in the future. (P11)*

Some parents felt a diagnosis would improve access to education support services and future planning as described here:

*Because he hasn’t ticked the autism box, school couldn’t get funding for him even though he finished year six at a year two level. So that’s been the main reason that I’m looking for a diagnosis because it can get help support for him and for us. (later) (a diagnosis will) give me an idea of maybe what’s possible, what might not be possible. I think it just helps because it helps us help him. (P12)*

This was particularly so for parents with older children who had experienced years of advocating for support within the education system.

*Getting him help and access to support is probably the biggest thing. In my view, that’s the most important thing instead of just ignoring him and letting him grow up illiterate and when he’s able to actually do these things, just being able to get support for him to help him and a name helps that. (P12)*

A number of parents spoke of the benefits that a diagnosis would mean not only for them, but for other families with a child similar to their own, including other families globally. This included parents whose dual purpose for participating in the program was in generating information for the benefit of other families, and potentially their own.

*Like you can’t make it better unless you have information, so that’s part of our, I think, our role in participating in this program is creating information to be used for others as well as our family’s benefit… it just creates information that can be used hopefully. (P5a)*

## Discussion

Through Spillmann et al.’s 2017 study, we are provided “a window into living with an undiagnosed disease” [17 p1] for parents and adult patients. In our study, the door has been opened and we have been welcomed into the homes of parents (figuratively and in some cases literally) to listen to their stories first hand. As a result, we see a much richer picture of parents on their journey and see that they occupy significant roles such as navigators, experts and advocates for their child. In this, they share similarities with the roles of parents of children with diagnosed rare conditions. Two Canadian studies [31, 32] explored the care coordination needs of children with diagnosed rare conditions and found non-integrated delivery of care gave rise to parents becoming advocates, case managers, and medical navigators for their children. Both studies [31, 32] found that parents developed expertise to the extent that they often knew more about their child’s disease than health care providers and that many parents would benefit from additional formal supports, including care coordination. The parents in our study share the roles of navigator, expert and advocate with parents of children with diagnosed rare diseases in many of the same ways. However, for our parents this occurs while a diagnosis is absent, adding a significant layer of complexity to their journey.

We also learn, of the many frustrations and hardships as parents and the medical profession make efforts to secure a diagnosis. Jutel and Nettleton [2] propose that the ‘rubrics’ of diagnosis comprise category, process and consequence. At its simplest, diagnosis is a linear progression where a diagnostic process is undertaken, a category is ascribed and then the patient, their family and care providers adjust and prepare for the consequence. Jutel and Nettleton [2] recognise the journey is frequently not straightforward and note that “when test results and clinical observations are not compatible, the diagnostician does not simply disregard her or his own assessment but undertakes repair work, at which point we begin to see the interface between diagnosis as a category and a process” [2 p795].

This interface between diagnosis as a category and a process is magnified in the diagnostic journeys experienced by our participants and is characterised by an almost never-ending friction of jarring movements between the two. Some parents insert themselves in the process stage of diagnosis by making suggestions to clinicians about the cause of their child’s condition. The tentative (possible) diagnosis and misdiagnoses received along the way, where parents are confronted with life-threatening and or limiting conditions and contemplate the consequences illustrates that our families, over the months and years of the diagnostic odyssey, are frequently experiencing the interface between all three domains of category, process and consequence. On top of this, parents are managing the extensive care needs of their children, in situations where symptoms can emerge suddenly or gradually over time and medical management and treatments change in a process of *trial and error.* It is no wonder that these journeys are described by our participants as a *rollercoaster* with significant emotional impacts and are easily recognisable as chaotic experiences [17].

Further, the meanings associated with diagnosis as a ‘category’ has been shaped by the journey our participants have travelled. On this journey an overarching, all-encompassing category (diagnosis) has been conspicuous by its absence. Along the way however, many families have acquired sub-categories (partial diagnoses) for aspects their child’s condition. A definitive, overall diagnosis is often viewed by parents as an opportunity to provide answers and a *context* which can cohere these sub-categories and the vast array of symptoms, and can dispel or confirm any tentative diagnoses.

While Jutel and Nettelton [2] recognise that diagnosis can be a “starting point” for sense-making, for our participants, it is anything but. A requirement to make sense for our parents, and those in other studies [33], is often needed from the moment health care concerns are raised about their child. While our parents convey a degree of “acclimation to illness uncertainty” [15 p7] that many people living with undiagnosed diseases develop, managing the uncertainty *eating us up* remains a significant issue. Compounding this is the need to *rattle cages*, to meet not only day to day care, but the diagnostic needs of their children, in some cases causing *burnout*. This, together with an inability to access the care pathways or support networks of those with similar conditions, reveals an alignment with chaos narratives [17, 18] and highlights a need for additional support in the absence of a diagnosis such as connection with, or local establishment of support groups for the undiagnosed such as Syndromes Without A Name (https://swanaus.org.au).

Offering some counterbalance to the chaos is the personal agency parents use through advocating and bringing expertise and the experience of personal growth. This is congruent with the ‘quest’ narrative described by Spillmann et al. [17] whereby parents of undiagnosed children are able to focus on the positive and discover new parenting strengths.

While some tension was reported by participant between themselves and the medical profession in relation to parents’ description of unusual symptoms experienced by their children, overall the medical profession recognised that children were living with an underlying illness of some kind. As such the need to seek recognition of the legitimacy of ill health from care professionals, a major feature of adults living with medically unexplained illnesses [14, 17], was largely absent in our interviews. Spillmann et al. [17], also found that the legitimacy of children’s undiagnosed illnesses was not a feature of the narratives of parents of a child with an undiagnosed condition (in contrast to probands), lessening an already difficult load for parents.

Parents in our study responded to the notion of diagnosis with pragmatism, altruism and a desire for connection. For some, pragmatism was reflected in an acceptance that their child has a condition that will be with them for life. This reflects ‘restitution’ as defined by Spillmann et al. [17] whereby there is an acceptance of the lifelong nature of a condition. The pragmatism expressed by our parents was also reflected in their desire for better management for their child. Further, they perceive that a diagnosis will guide expectations and treatment; parents are searching for a category which “offers explanations and coheres patient symptoms” [2 p793]. Additionally, parents sought connection to other families (other ‘experts’), primarily from the viewpoint that these connections may offer opportunities to better manage their own child’s care.

The difficulties experienced along the diagnostic journey, particularly those related to an absence of a diagnosis, provide important context for the values parents’ place in the potential outcomes of the UDP-WA. While remaining conservative in their expectations of the likelihood of a diagnosis and what it may deliver, all parents did see value in a diagnosis—it would be welcomed rather than eschewed [2]. The perceived value in diagnosis to guide expectations and treatment [7] as well as seeking community-based supports has been found in various other studies [9, 33]. As has parents seeking a diagnosis to better understand their child’s condition and behaviour and to help them to acknowledge and plan the life-long care needed for their child [33, 34]. In addition to the benefits the UDP-WA families may obtain, many parents expressed altruism in that they hoped their participation in the program will provide benefits for other families.

## Conclusion and policy implications

Our study with parents of children in the UDP-WA shows that families tend to experience very difficult diagnostic odysseys. These are characterised by the demands faced by many parents of children with diagnosed rare diseases, yet are overlaid with many additional challenges related to lack of diagnosis. Nonetheless, many parents report making adaptation and experience personal and family growth along the way. At the commencement of the UDP-WA while carefully moderating their expectations of a diagnosis and its consequence, parents perceive significant value in receiving a diagnosis for their child. Our study highlighted that parents, through the difficulties in their experiences on the diagnostic odyssey, and in their roles as ‘navigator, expert and advocate’, may have unmet needs that fall beyond the scope of the UDP-WA.

Accordingly, a number of policy implications emerged. Firstly, there is a need to explore the ways in which systemic barriers to timely, accurate diagnosis can be minimised or removed. In particular opportunities should be explored to improve multidisciplinary team care, by addressing known systemic limitations such as the way medical disciplines are siloed in health systems. In developing such opportunities to meet unmet needs in diagnosis and care, it is important to recognise the common experiences of parents of a child with an undiagnosed disease and a diagnosed rare disease, particularly with respect to ongoing care needs. Policies and programs that support one will often support the other. For example, care coordination programs can benefit families of children with both diagnosed rare diseases and undiagnosed conditions. It is essential that development of programs should be explored with a focus on access that is needs based, not predicated on a diagnosis.

Some parents in the study spoke of the value in the support group to which they belonged or approached for support; however other parents wanted but did not have access to such groups. Peak organisations, such as Australia’s national peak organisation for rare diseases, Rare Voices Australia, have a role to play in strengthening existing peer support organisations, and fostering new organisations where gaps exist. In the context of undiagnosed diseases, while there is an Australian-based chapter of Syndromes Without A Name (https://swanaus.org.au), there is no Western Australian chapter or significant local profile. The findings from this study suggest value in exploring opportunities to improve local access to such support groups.

Finally, the report produced at stage 7 of the UDP-WA, should, where practicable, present complex medical information about a diagnosed or still undiagnosed child that is meaningful in non-medical care systems. For example, a report that considers the child and families’ movement through school and disability services and helps to inform the care needs in those systems, has the potential to afford great benefit to families.

## Limitations

There are a number of limitations that need to be considered in our study. The sample size was small and the care needs of the children varied considerably. However, the reflections from parents illustrated the shared experiences of the diagnostic odyssey for families of a child with an undiagnosed condition, at the point when they have agreed to participate in the UDP-WA. Nonetheless, the experiences may not be representative of other families with children living with undiagnosed conditions. The sample size also limited our ability to capture socio-demographic data and cultural background (such as Indigenous Australians or people from culturally and linguistically diverse backgrounds) and maintain anonymity. Further, with the exception of one male participant, our sample was all female, which limits the voice of fathers and male carers in this study.

## Data Availability

Data sharing is not applicable to this article as no datasets were generated or analysed during the current study.

## Footnotes

1 In Australia, the disability services sector provides speech, occupational and physio therapies as well as supports like respite care.
